# Adaptive fabric with emissivity regulation for thermal management of humans

**DOI:** 10.1515/nanoph-2023-0930

**Published:** 2024-06-03

**Authors:** Xiansheng Li, Meiling Liu, Ken Chen, Lanxin Li, Gang Pei, Bin Zhao

**Affiliations:** Department of Thermal Science and Energy Engineering, University of Science and Technology of China, Hefei 230027, China; National Synchrotron Radiation Laboratory, School of Nuclear Science and Technology, University of Science and Technology of China, Hefei, Anhui 230029, P.R. China

**Keywords:** emissivity modulation, smart fabric, radiative heat dissipation, silver nanowires

## Abstract

The heat generation of the human body dramatically varies between resting and active status, so dynamic heat dissipation is required to ensure optimal thermal comfort. Herein, we propose a spectrally self-adaptive smart fabric (SSSF) by covering polyester fabric with silver nanowires, which autonomously adjusts its emissivity in response to the body’s movement status from dry to wet states. During periods of inactivity, the SSSF maintains radiative heat insulation with a low emissivity state of 0.39. Conversely, during vigorous physical activity, its emissivity is improved to 0.83 when the sweat penetrates the SSSF, facilitating greater heat dissipation. Comparative experiments demonstrate the superior thermal management capabilities of the SSSF, with a 19.5 % reduction in heat dissipation power relative to traditional fabrics when in the low emissivity mode, and an impressive 67.6 % enhancement in heat dissipation power as it changes from low to high emissivity mode. This work provides an adaptive approach to emissivity modulation, offering an effective solution for dynamic heat dissipation of humans across various states of activity, thereby enhancing personal thermal comfort.

## 1 Introduction

The thermal comfort of the human body is a widely discussed topic as it significantly impacts our physiological and psychological health [1], [2], [3], [4]. Generally, humans strive to maintain a suitable temperature range in diverse environmental conditions to ensure regular physiological function [5], [6], [7], [8]. For example, during physical exercise, humans’ muscles become highly active and generate a significant amount of heat, which necessitates effective heat dissipation to maintain body temperature within a normal range. Conversely, when inactive, especially in cold environments, reducing heat dissipation of the body is desirable to keep the body warm. To meet the above demands, it is crucial to properly regulate the heat transfer process between the human body and its surroundings [9].

Fabric plays a critical role in managing the thermal comfort of the human body since it acts as a medium for heat exchange between the human body and surroundings [10], [11], [12], [13], [14]. More than 50 % of the human body’s thermal energy is released into the environment through thermal radiation [15]. Therefore, fabric with tailored spectrums can effectively affect the heat dissipation of the human body, thereby improving thermal comfort. In recent years, emissivity engineering techniques have been developed rapidly [16], [17], [18], [19], [20], and two kinds of advanced fabrics, radiative cooling fabric and thermal radiation insulation fabric, have garnered interest in personal thermal comfort regulation by enhancing and reducing the thermal emissivity of the fabric layers [21], [22], [23], [24]. For example, Cui et al. developed composite textiles with spectral selectivity for passive radiative cooling by adding zinc oxide nanoparticles to nanoporous polyethylene [25], which can achieve a fabric’s temperature reduction of 8 °C compared to cotton. Zhou et al. sprayed Ti_3_C_2_T_*x*_ on the surface of the fabric to reduce the emissivity of the fabric surface to 0.195 at 7 µm–14 µm. The temperature of the human skin covered with the fabric is 2.68 °C higher than that covered with cotton fabric [26]. Although the aforementioned fabrics contribute significantly to human thermal management by providing mechanisms for enhanced and suppressed radiative heat dissipation, their spectral properties are static, which means these fabrics are monofunctional to keep humans cool and warm. However, the heat dissipation requirements of humans under resting and physical exercise are quietly different, which need dynamical thermal management for thermal comfort. Therefore, the concept of smart fabrics with switchable emissivity modulation for self-adjusting radiative heat dissipation enhancement and suppression has been proposed and developed [27], [28], [29], [30]. Cui et al. developed a smart fabric by sandwiching two nanoPE layers together, one is covered with a copper layer and the other is coated with carbon. This fabric exhibits an average emissivity of 0.894 on the carbon-coated side, while the side with the copper coating has an emissivity of 0.303 [30]. Similarly, Gao et al. introduced a dual-sided intelligent fabric using polyimide (PI) and Ag nanowires (AgNWs), which shows high reflectivity of 0.80 on the AgNWs coated side and strong infrared emissivity of 0.79 on the PI side [31]. However, these fabrics often require active mechanical flipping to switch the different sides of the fabrics to achieve emissivity modulation, which is not friendly for the end user. Aiming at this point, this work plans to propose a passive strategy to achieve dynamic emissivity modulation of the fabric, which is quite different from the above mentioned Janus fabric with active operation.

In this work, we introduce a spectrally self-adaptive smart fabric (SSSF) by covering a highly breathable polyester fabric with a network of AgNWs for the thermal management of the human body. The emissivity of the SSSF can be passively controlled in response to the body’s movement status from dry to wet states, dynamically achieving radiative heat dissipation enhancement and suppression for humans at different activity levels. During resting status, the SSSF maintains a low-emissivity state (*ε* = 0.39) to suppress radiative heat loss from the body to surroundings, while it changes to a high-emissivity state (*ε* = 0.83) when the sweat penetrates the SSSF to enhance radiative heat dissipation during exercise. Experimental demonstration indicates that the SSSF at the low-emissivity state can save heat loss power of 19.5 % compared to the traditional commercial fabric. Besides, the heat dissipation power of the simulated skin is enhanced by 67.6 % when the SSSF switches from low-emissivity mode to high-emissivity mode coupled with evaporation cooling. These results underscore the significant potential of the SSSF for passive thermal management of the human body.

## 2 Results and discussion

### 2.1 Fabrication and characterization of the SSSF

**Figure 1:**
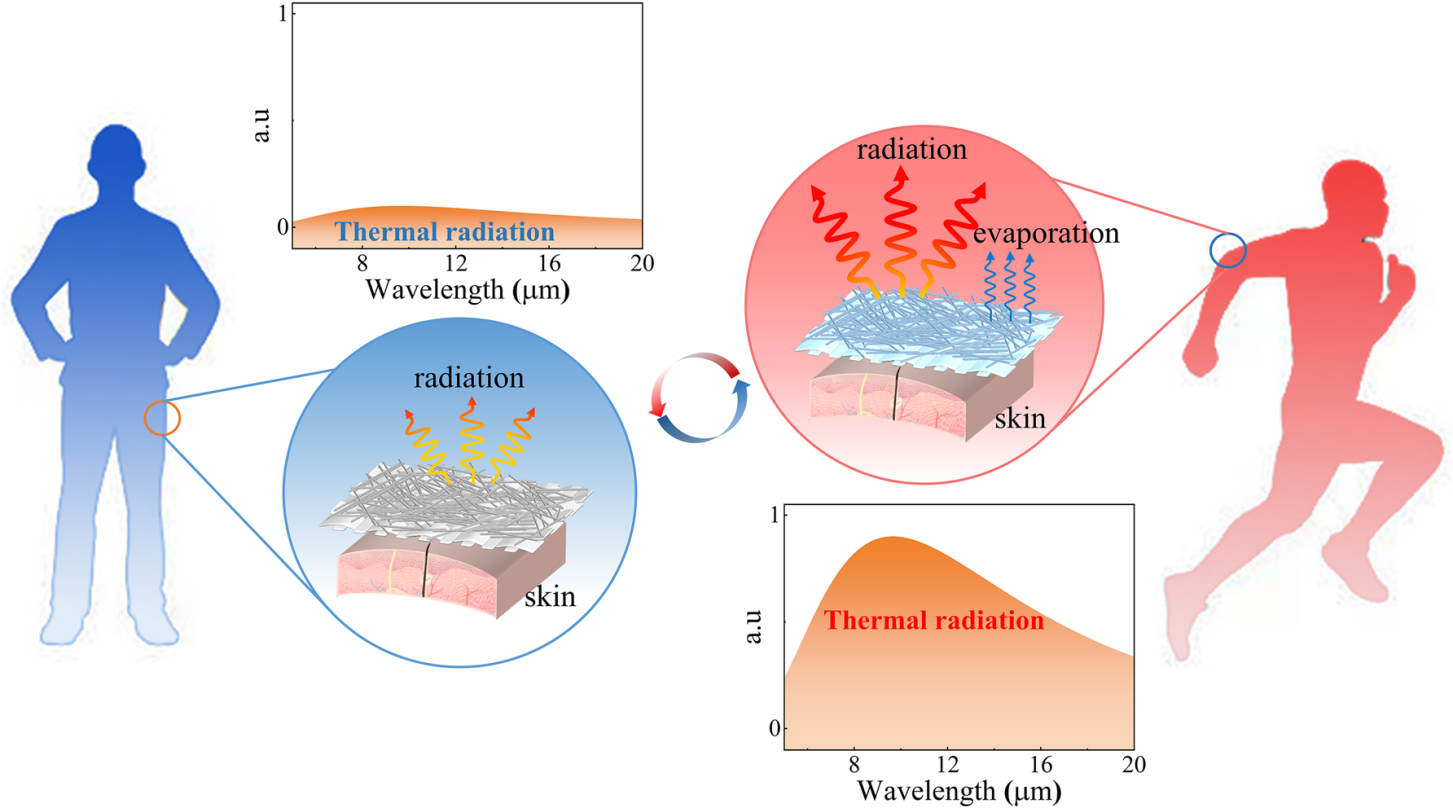
Thermal management schematic of the SSSF under resting and physical activity status of the human body.

In a resting status, the human body produces a heat output of approximately 70 W/m^2^ [32], [33]. In cold environments, employing low-emissivity fabrics is essential to minimize the radiation heat loss from the body and maintain thermal comfort. Conversely, during physical activity, the heat output of the human body can exceed 150 W/m^2^ [34], [35], making fabrics with high emissivity beneficial for the dissipation of excess heat. Therefore, fabrics with adjustable emissivity provide benefits in maintaining thermal comfort for the human body before and after physical activity. Based on this consideration, we propose a strategy for passive emissivity modulation (Figure 1). The fabric needs to have low emissivity at resting status (i.e., low-emissivity state) and this can be achieved by covering a reflecting coating on top of the fabric, while the fabric needs to transmit the sweat and change it to be high emissivity at physical activity status (i.e., high emissivity state).

During the fabrication process, AgNWs are utilized as a porous low-emissivity coating on fabrics. These AgNWs self-assemble into a network on the surface of the fabric, achieving a high reflectivity (low emissivity) due to the small distances between wires, which are significantly less than the wavelength of mid-infrared light. Moreover, the polyester fabric with small interstitial spaces is selected as the substrate to enhance the surface area available for AgNW attachment and to reduce the loss of AgNWs from the fiber gaps during the coating process. Additionally, the gaps between fibers of the polyester fabric facilitate sweat penetration from the skin to the exterior surface of the AgNWs, thereby enabling a high emissivity state. Of course, the permeability characteristic is one of the characteristics of most polyester fabrics.

**Figure 2:**
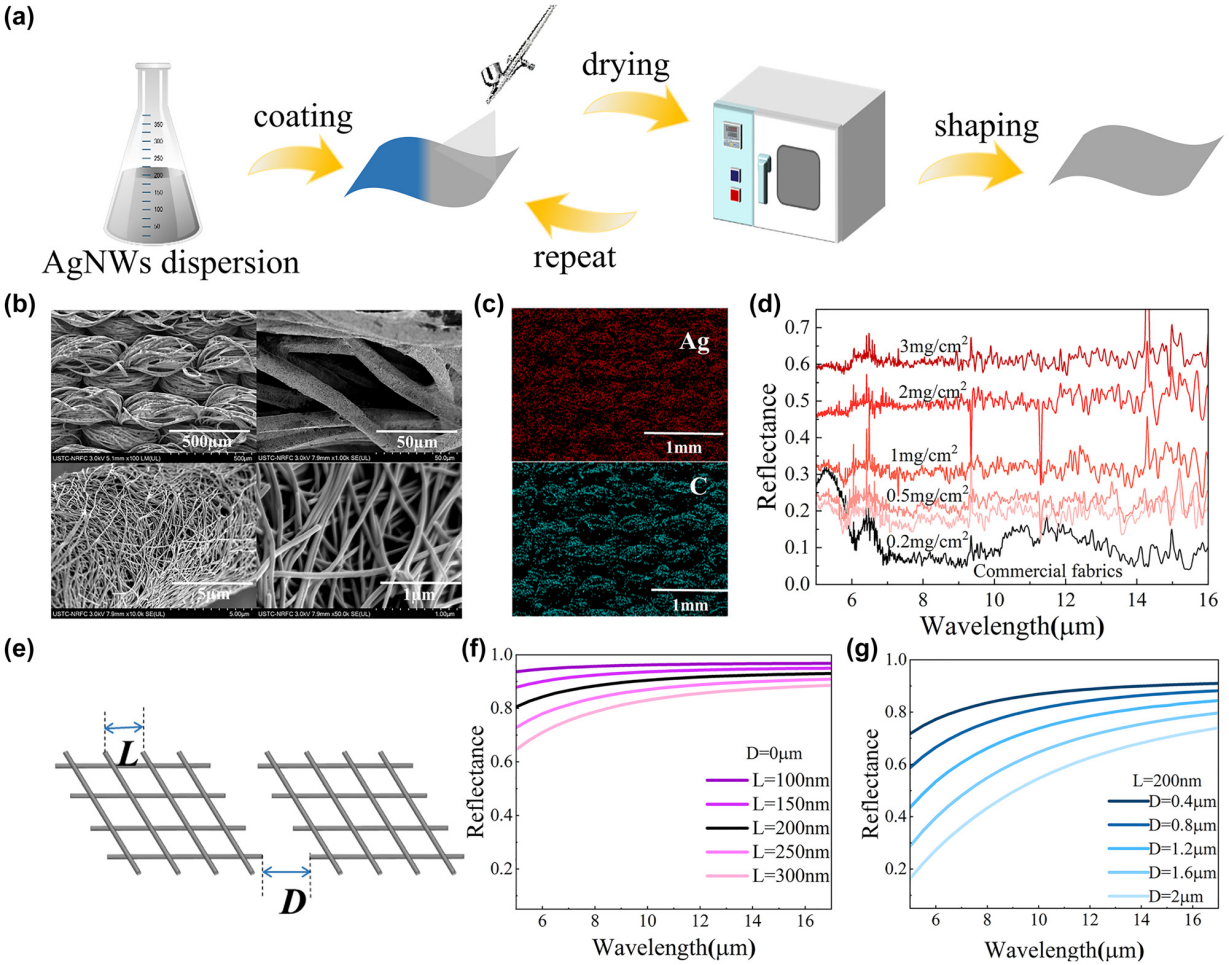
Preparation and characterization of the SSSF. (a) The fabrication process of the SSSF. (b) SEM image of the SSSF. (c) The EDS image of SSSF. The distribution of Ag and C elements is shown. (d) Spectral reflectivity of both commercial fabrics and commercial fabrics coated with different concentrations of AgNWs. (e) An optical model of the AgNWs metal network. *L* denotes the distance between adjacent AgNWs, while *D* represents the gap between AgNWs grids. (f) Spectral reflectivity of the AgNWs network when the distance between adjacent AgNWs ranges from 100 nm to 300 nm under *D* = 0 µm. (g) Spectral reflectivity of the AgNWs network when the distance between AgNWs grids spans from 0.4 µm to 2 µm under *L* = 200 nm.

The fabrication process of the SSSF is depicted in Figure 2a. First, the AgNWs solution is diluted and sprayed onto the surface of the commercial polyester fabric. Subsequently, the fabric is dried in an oven. This spraying and drying process is repeated until all the AgNWs are fully coated onto the fabric surface. As shown in Figure 2b, the AgNWs sprayed onto the fabric adhere to the polyester fibers and AgNWs exhibit a mesh-like distribution. Besides, it can be found that the AgNWs encase the polyester fibers. To prove the AgNWs have successfully coated onto the polyester fabric surface, EDS characterization is further conducted and is shown in Figure 2c.

The infrared reflectivity of the fabric is improved when AgNWs are applied and this reflection is further enhanced by enlarging the mass concentration of AgNWs (Figure 2d). This improvement is attributed to the denser coverage of AgNWs on the fabric surface as the concentration of AgNWs increases, which results in a corresponding increase in reflectance. Notably, although the infrared reflectivity is enhanced by high-concentrated AgNWs, the excessive density of AgNWs compromises both their adhesion to the fabric and leads to an escalation in costs. Hence, a mass density of 3 mg/cm^2^ for AgNWs is applied for the SSSF, achieving a reflectivity of 0.61, which corresponds to an emissivity of 0.39 based on the fact that the SSSF is infrared opaque.

To describe the infrared characteristics of the SSSF under the low emissivity state, an optical simulation is performed based on a simplified silver metal grid model that is used to simulate an equivalent AgNWs grid. It can be observed from the SEM image that the AgNWs network is dispersed on the surface of the fabric. However, due to the coarse fibers of the fabric, the AgNWs network is not continuous and is segmented by the fibers of the fabric (Figure 2b). Therefore, the AgNWs network on the fabric surface is composed of numerous tiny AgNWs networks. Figure 2e shows the equivalent silver metal network model, where *L* represents the distance between adjacent silver metal wires and *D* represents the distance between adjacent silver metal tiny networks. As *L* increases from 100 nm to 300 nm, the reflectivity (at the wavelength of 10 μm) of continuous silver metal networks (*D* = 0 mm) decreases from 0.95 to 0.8 (Figure 2f). Evidently, the simulated results of the continuous metal network significantly exceed the actual reflectivity. This is because the fabric surface is high roughness and the physical segmentation of silver nanowire networks by the polyester fibers in the fabric. To further describe the spectral characteristics of the fabric surface, the reflectivity of adjacent tiny metal networks at different *D* is simulated. When *L* = 200 nm, as the *D* increases from 0 µm to 2 µm, the infrared reflectivity (at the wavelength of 10 μm) decreases from 0.9 to 0.55 (Figure 2g).

**Figure 3:**
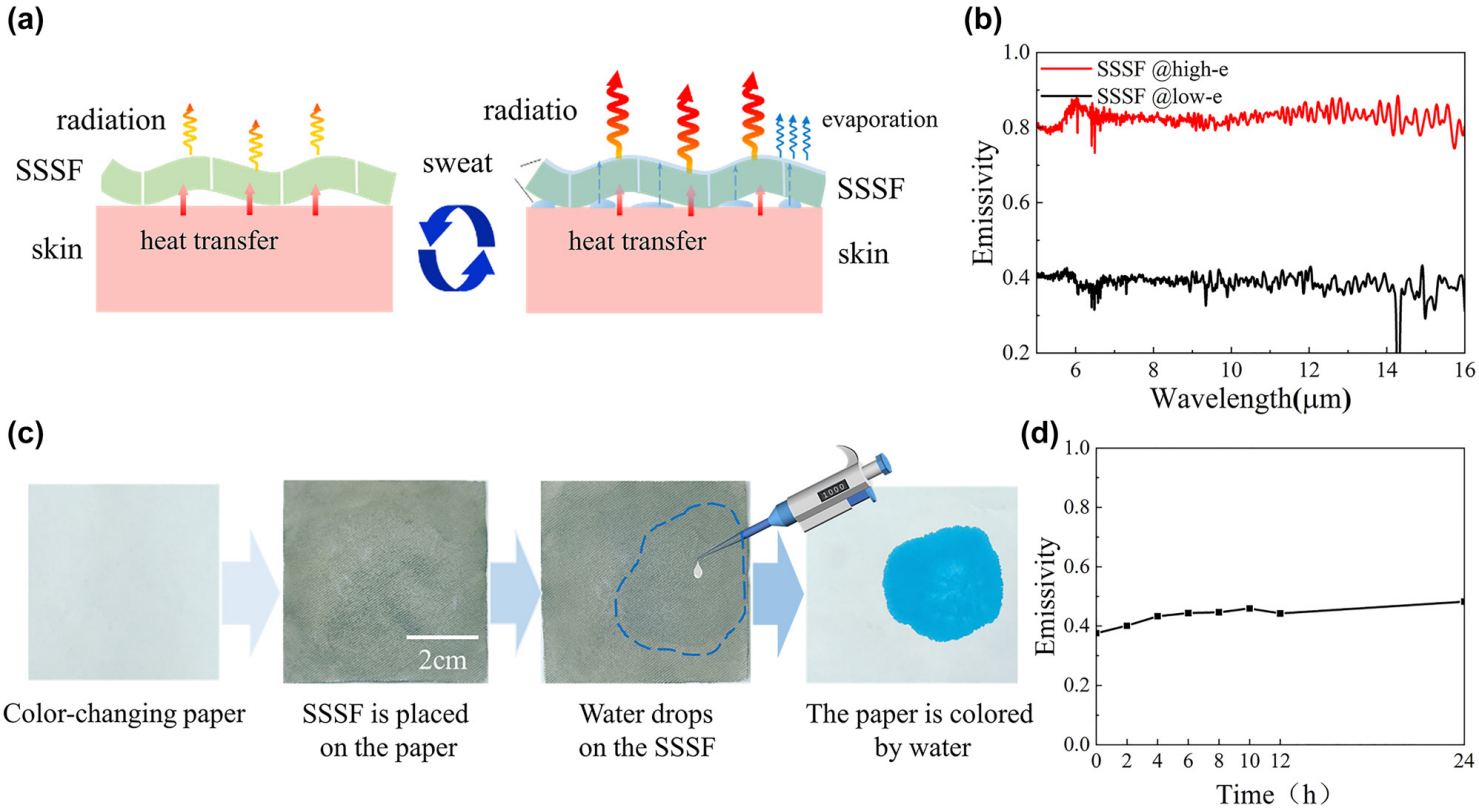
Spectral control mechanism and performance of the SSSF. (a) Thermal management process of the SSSF. (b) Spectral emissivity of the SSSF under different modes. (c) Evaluation of the water penetrative performance of the SSSF. (d) Spectral emissivity of the SSSF after water washing operation.

During resting status, the fabric can preserve a state of dryness, which exhibits a low emissivity surface due to the metallic characteristics of AgNWs. This feature suppresses radiative heat loss from humans. However, during physical activity with sweat, the sweat penetrates the fabric and adheres to the exterior surface of the SSSF. Due to the high emissivity characteristic of water, the SSSF exhibits enhanced radiative properties (Figure 3a). Simultaneously, as the sweat evaporates from the fabric surface, it also carries away a portion of the generated heat. The combined effect of radiative and evaporative heat dissipation contributes to the strong heat release of the human during intense physical activities. Consequently, the SSSF exhibits an emissivity of 0.39 under a dry state, while the emissivity rises to 0.83 under a wet state, achieving an emissivity modulation of 0.44 (Figure 3b). The sweat penetrative capability of the SSSF is crucial in modulating the emissivity. As shown in Figure 3c, to demonstrate the sweat-penetrative property of the SSSF, we place the SSSF on a color-changing paper that turns blue upon contact with water. When water drips on the fabric, the water passes through the SSSF, and the paper underneath turns blue. The interstices between the polyester fibers in the fabric, as well as the gaps between the AgNWs networks, are far larger than the diameter of water molecules, thereby facilitating the permeation of water through these gaps. The permeability of fabric to water depends on the characteristics of polyester fabric, and AgNWs has little effect on the permeability of fabric. Therefore, the evaporative cooling performance of SSSF is minimally affected compared to ordinary polyester fabrics [36]. Furthermore, to assess the wear resistance and water resistance, the SSSF is placed in water and continuously stirred for 24 h. Measured spectral emissivity (Figure 3d) shows that the emissivity is only attenuated by 0.1.

### 2.2 Thermal performance of the SSSF

**Figure 4:**
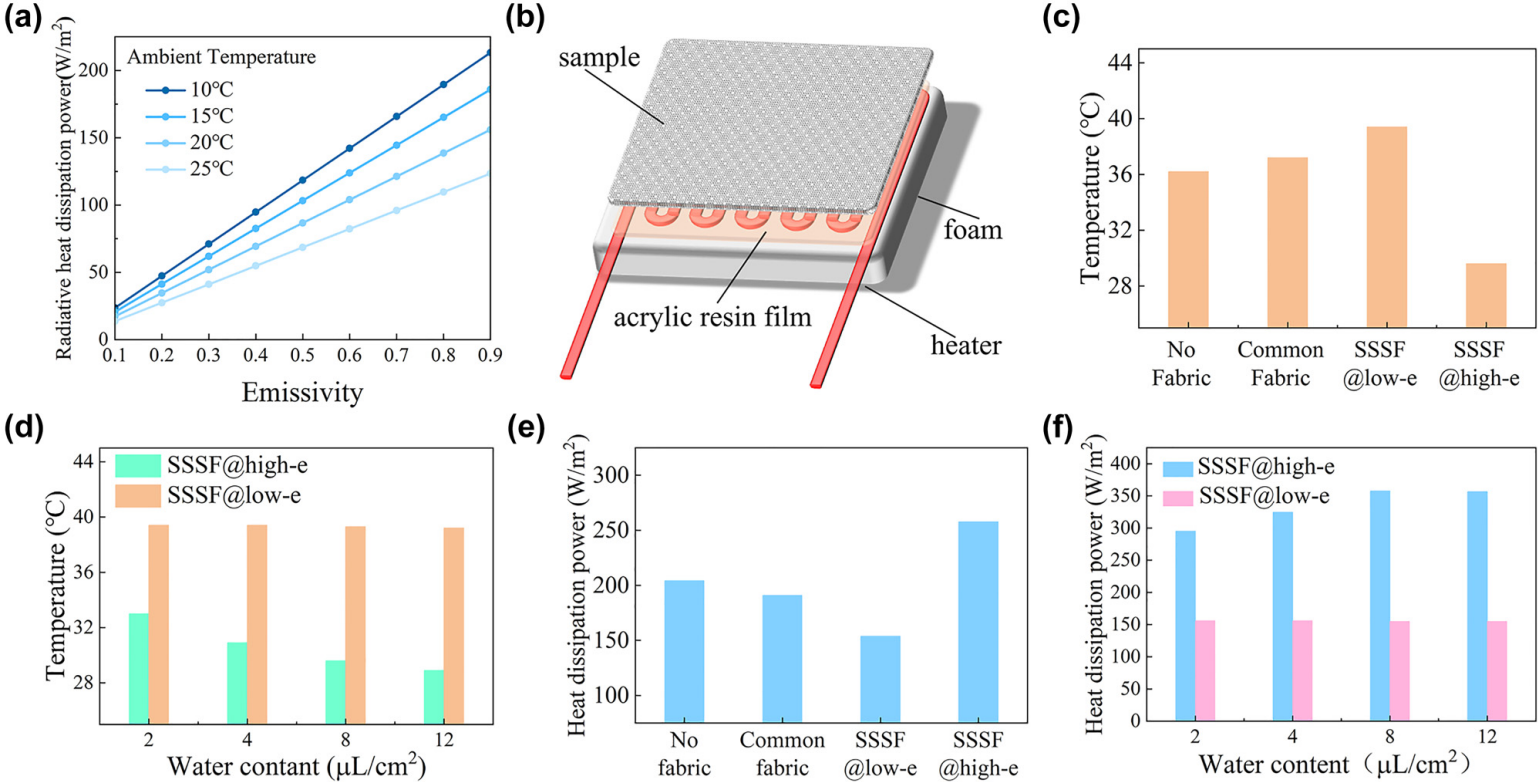
Thermal performance of the SSSF. (a) Calculated radiative heat dissipation from the clothing surface into the surrounding environment at varying emissivities and ambient temperatures. (b) Simulated skin experimental apparatus for assessing the thermal physical properties of fabrics. (c) Stagnation temperature evaluation of the simulated skin with various coverings under a constant input power. (d) Assessment of the skin temperature covered with the SSSF under different water mass densities at a constant input power. (e) Comparative evaluation of heat dissipation of the simulated skin with various coverings, while maintaining a constant skin temperature. (f) Assessment of the heat dissipation of the simulated skin covered with the SSSF at different water mass densities, while maintaining a constant skin temperature.

The surface emissivity of human clothing plays an important role in the efficiency of radiative heat dissipation. Figure 4a displays the calculated radiative heat dissipation power of the fabric at varying surface emissivities and different ambient temperatures based on Eqs. (1) and (2). Under an ambient temperature of 20 °C, when the emissivity of the outer surface of the clothing is 0.1, the radiative heat dissipation power is merely 17 W/m^2^. However, when the emissivity is improved to 0.9, the radiative heat dissipation power reaches 156 W/m^2^. The radiative heat exchange between clothing and the surrounding environment can be expressed by the equation

(1) $$q=\varepsilon \sigma \left(T_{\text{cl}}^{4}-T_{\text{sky}}^{4}\right)$$

(2) $$T_{\text{sky}}=0.0552{T_{a}}^{1.5}$$

where *ε* is fabric emissivity, *σ* is the Stefan–Boltzmann constant, *T*_cl_ is the temperature of the fabric surface, *T*_sky_ is the effective sky temperature, and *T*_*a*_ is the ambient air temperature.

Subsequently, the heat preservation and dissipation functionalities of the SSSF fabric are evaluated by measuring the surface temperature and heat dissipation power in wet (high-emissivity) and dry (low-emissivity) states using a simulated skin device. As shown in Figure 4b, the device consists of a low thermal conductive foam board, a heating element, and a layer of polypropylene resin film. The low thermal conductive foam is used to prevent heat loss from the bottom. The heating element is used to simulate the internal heat source of the human body and the polypropylene resin film is used to simulate human skin because polypropylene resin has a similar emissivity to human skin [30]. A thermocouple is placed on the surface of the polypropylene resin to monitor the surface temperature of the simulated skin.

As shown in Figure 4c, the surface temperatures of the simulated skin under a fixed heating power are recorded and compared across different coverings: commercial fabric, SSSF at the low-emissivity state, SSSF at the high-emissivity state, and without anything. The simulated skin covered with the low-emissivity SSSF exhibits the highest temperature, which is 2.2 °C and 3.2 °C higher than the commercial fabric-covered skin and bare skin, respectively. In contrast, the simulated skin covered with the high-emissivity SSSF is 6.6 °C lower than the skin covered with the low-emissivity SSSF, showing that the emissivity of the fabric closely affects the skin temperature and thermal comfort of humans. Considering that the human body’s heat production rate and sweat rate increase in proportion to the intensity of physical activity, we further investigate the impact of different levels of perspiration on simulated skin temperature by dripping different masses of water onto the front side of the fabric and monitoring the skin temperature. It can be seen from Figure 4d that the stagnation temperature of the simulated skin gradually decreases and reaches a steady state as the mass of water per unit area increases. This is because as the mass of water per unit area increases, the wetted area of the SSSF expands, leading to an enhancement of radiative heat dissipation. When the SSSF is fully wetted, radiative heat dissipation reaches a relatively stable level.

Additionally, the heat dissipation power of the simulated skin is also measured across various fabric coverings under a constant skin temperature of 36 °C (Figure 4e). Compared to common fabric and bare skin, the SSSF with the low-emissivity state reduces the heat dissipation power by 37.2 W/m^2^ and 50.4 W/m^2^, respectively, leading to reductions of 19.5 % and 24.6 %. Conversely, the heat dissipation power of the SSSF increases by 104 W/m^2^, up by 67.6 %, when the SSSF changes from the low-emissivity state to the high-emissivity state. Similar to Figure 4d, as the mass of sweat per unit area increased, the maximum heat dissipation power also increased (Figure 4f). The above set of experiments reveals that the SSSF has the potential to dynamically control the heat dissipation of humans based on the sweat stimulus.

**Figure 5:**
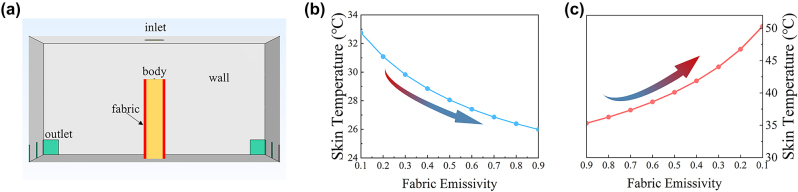
Effect of fabric surface emissivity on human body thermal comfort. (a) A 3D model constructed for simulation of human body temperature in a controlled environment. (b) The variation of skin temperature of humans at resting status with emissivity variation. (c) The variation of skin temperature of humans during physical activity with emissivity variation.

To further analyze the effect of fabric emissivity on human comfort, we develop a 3D computational model for temperature simulation. As illustrated in Figure 5a, the room dimension is 6 m × 6 m × 3 m, and it has one inlet and twelve outlets to ensure sufficient air circulation within the space. We simulate two scenarios representing resting and exercising states, with a body heat generation rate of 70 W/m^2^ and 200 W/m^2^, respectively. In the resting status, assuming an initial fabric emissivity of 0.1, the airflow velocity entering the room is set to maintain the skin temperature at about 33 °C [37], [38]. As the emissivity of the fabric surface increases, the temperature of the skin surface decreases by 4 °C (Figure 5b), indicating that the low-emissivity surface has a radiative thermal insulation effect (Figure 5b). In the exercising state, assuming an initial emissivity of 0.9 and a body heat generation rate of 200 W/m^2^, the inlet airflow velocities are set to maintain the skin temperature at about 35 °C [37], [38] and the other parameters remain unchanged. As the emissivity of the fabric surface decreases, the skin temperature rises by about 15 °C, revealing that high-emissivity fabric can enhance radiative heat dissipation during exercise (Figure 5c). Overall, this simulation aims to show that the emissivity of a fabric surface can significantly alter radiative heat dissipation of humans. Specifically, fabric with the low emissivity state can make humans warm during resting, while fabric with the high emissivity state can make people feel cool during exercise.

## 3 Conclusions

In this work, we have designed a spectrally self-adaptive smart fabric (SSSF) based on polyester fabric and AgNWs, which can dynamically regulate its surface emissivity in response to sweat, benefiting the smart thermal management of the human body across varying levels of physical activity. During the resting period, the SSSF remains dry with an infrared emissivity of 0.39, effectively suppressing radiative heat loss and keeping warm. Conversely, during intense physical activity, sweat penetrates to the exterior surface of the SSSF, resulting in a high emissivity of 0.83, facilitating enhanced radiative heat dissipation and keeping cool. Indoor experiments have demonstrated that the heat dissipation power of the SSSF is 19.5 % lower than that of commercial textiles in resting status under a fixed skin temperature. However, when changing to physical activity, the heat dissipation power of the SSSF is increased by 67.6 % based on enhanced radiative and evaporative heat dissipation. Overall, this study introduces a straightforward and cost-effective approach to improving the thermal comfort of the human body during both active and resting status based on self-adaptive emissivity modulation engineering, showing potential in the fields of personal thermal management.

## 4 Experimental section

### 4.1 Preparation of the SSSF

Commercially fabric that is composed of 100 % polyester is selected as the substrate. The AgNWs solution with a concentration of 5 mg mL^−1^ (Jiangsu Xianfeng Nano Material Technology Co.) consists of silver nanowires with a diameter of 30 nm and a length of 20 μm dispersed in ethanol. The AgNWs solution is first diluted to a concentration of 0.5 mg mL^−1^ and subsequently sprayed onto the fabric substrate using a spray gun, followed by the drying process in an oven. The manipulation of various mass ratios of the AgNWs in the SSSF is controlled by adjusting the volume of the diluted solution used for spraying and the number of spray operations.

### 4.2 Characterization of the SSSF

The spectral reflectivity and transmittance of the SSSF with the mid-infrared wavelength band is measured by a Fourier transform infrared spectrometer (Nicolet iS 10, Thermo Scientific) equipped with a gold-coated integrating sphere (PIKE). Then, the spectral emissivity of the SSSF is calculated based on the energy balance law and Kirchhoff’s law. The surface morphologies, cross-section images, and element distribution of the SSSF are measured by Scanning Electron Microscopy (SEM, FESEM SU8220, Hitachi).

### 4.3 Thermal performance measurement of the SSSF

Thermal performance testing of the SSSF is conducted in indoor environments, with air temperature controlled at 24 °C. During the temperature measurement experiment, the first step is applying an appropriate fixed power to the bare simulated skin (polypropylene resin) to keep the skin temperature at 36 °C. Subsequently, at the same power level, the skin temperature is measured with different fabric coverings. In the power testing experiment, the heat dissipation power test results are obtained through the feedback method. Thermal compensation technique is utilized to hold the skin temperature at 36 °C and the input power is monitored under different fabric coverings. The feedback heating power is recorded by the integrated testing and data acquisition device (TP700). T-type thermocouples with an accuracy of ±0.5 °C are applied for temperature measurement.

### 4.4 Computational fluid dynamics (CFD) modeling

The simulation is performed using COMSOL 6.1. The entire domain uses hexahedral cutting element meshes with a total number of 125,840. An achievable *k*–*ε* model with a standard wall function is used to handle turbulence.
